# Invasion strategies in round goby (*Neogobius melanostomus*): Is bigger really better?

**DOI:** 10.1371/journal.pone.0190777

**Published:** 2018-01-05

**Authors:** Joerg Brandner, Alexander F. Cerwenka, Ulrich K. Schliewen, Juergen Geist

**Affiliations:** 1 Wasserwirtschaftsamt Regensburg, Regensburg, Germany; 2 Aquatic Systems Biology Unit, Technical University of Munich, School of Life Sciences Weihenstephan, Freising, Germany; 3 SNSB-Bavarian State Collection of Zoology (ZSM), München, Germany; Xiamen University, CHINA

## Abstract

Few studies have systematically investigated mid- or long-term temporal changes of biological characteristics in invasive alien species considering the different phases of an invasion. We studied the invasion performance of one of the most invasive species worldwide, the round goby *Neogobius melanostomus*, from total absence over first occurrence until establishment from 2010 to 2015 in the upper Danube River. After an upstream movement of the invasion front of about 30 river km within four years, the pattern that round goby pioneering populations significantly differ from longer established ones has been confirmed: Pioneering populations at the invasion front comprised more females than males, and adult specimens with a larger body size compared to those at longer inhabited areas. On the population-level, the proportion of juveniles increased with time since invasion. The results of this study provide support for the previously postulated ´bigger is better´ and ´individual trait utility´ hypotheses explaining invasion success in round goby. Pioneering invaders with their greater exploratory behavior, highly adaptive phenotypic plasticity and increased competitive ability seem to act as prime emperors of new habitats, strongly following and benefiting from man-made river-bank structures.

## Introduction

Biological invasions are highly complex processes consisting of different stages, each with an independent probability of failure [1]. ´Introduction´, ´establishment´ and ´spread´ have long been differentiated as the characteristic phases of a biological invasion (e.g., “community assembly”-theory [2], “integrated conceptual model” [3]). Kolar and Lodge [4] added an ´impact´-phase to these concepts. To date, the terminology referring to these phases (stages) are common sense in invasion biology. Blackburn et al. [5] proposed a ´unified framework´ for biological invasions that is characterized by a consecutive series of phases with barriers that need to be overcome for a species or population to pass on to the next stage. However, there is a lack of field monitoring data that has critically validated this sequence in nature. Moreover, assigning field data to distinct phases of an invasion process is difficult. This is especially true between establishment and spread which often comprises an intermediate lag-phase, i.e. a latency period in the early stages of exponential population increase [3]. Consequently, nearly every single species can potentially become invasive as soon as introduced into another bio-geographical region [6,7]. However, it still remains unknown, why only few species manage to successfully spread and become invasive with severe adverse impact on other biota, as well as economically. Successful invaders are clearly not a random selection of species [8] as evident from an over-proportional percentage of vertebrates among invasive alien species (IAS) [9,10].

“*Propagule pressure*” is considered an important variable explaining invasion success [9,11], representing a composite measure of inoculation size (number of individuals released) and propagule number, i.e. the number of discrete release events into a new environment [11]. However, specific characteristics of propagule itself have hardly been examined [12]. Characteristics of pioneering invaders may play a key role in determining invasion success as suggested by different abundances of individuals carrying outlying biological traits during the different phases of an invasion [13].

In case of plants, an *“evolution of increased competitive ability”* has been proposed, suggesting that invaders produce more seeds or grow more vigorous and taller in environments outside their native ranges [14]. Analogously, this concept appears applicable to invasive animals, since pioneering invaders were reported to differ from their conspecifics in longer established populations by greater body sizes and condition factors, reduced parasitic load or different feeding strategies [15,16]. Particularly in the early phases of an invasion process, plasticity in life history traits seems to result in an important advantage related to invasion success [16,17], allowing for rapid adaptation to different environments [18–22]. Worldwide, the most successful aquatic invaders are also those that create the most serious ecosystem impacts [23], with invasive species being among the most potent drivers of global biodiversity loss [23–25].

To date, more than 140 non-native aquatic species are known from German water ways, with approximately 20% of these being invasive [26]. Due to both intentional and unintentional introductions by shipping, (man-made) waterway interconnection, ornamental trade and stocking action, introduction rates of aquatic IAS have highly accelerated over the last decades [26]. During the Joint Danube Survey 3, in the upper section of the Danube River, 24 macroinvertebrate IAS were recorded with a mean contribution to the total relative abundance of 49% in all benthic invertebrate species and 36% in fishes [27].

Over the last two decades, the round goby *Neogobius melanostomus* (Pallas, 1814), a benthic Ponto-Caspian gobiid fish (Teleostei: Gobiidae), has colonized both freshwater and marine ecosystems on both sides of the Atlantic Ocean outside its natural distribution range [28]. With increasing numbers of rapid range expansions, *N*. *melanostomus* invasions have been reported from the Laurentian Great Lakes watershed [28–31], from almost the entire Baltic Sea region [32–34] and from many other large European waterbodies, including the Rivers Oder, Elbe, Weser, Rhine [35–37] and the Danube [13,17,19,38–40]. Its ongoing rapid spread and its high potential to cause ecological regime-shifts (e.g. [41–44]) have accelerated substantial scientific interest in this species worldwide (reviewed in Kornis [29]). Studies on this species have focused on sampling methods [45], feeding ecology [19], parasitology [46], genetics [47], morphology [38], reproduction [48], plasticity in life history traits [17], individual performance [13] and range expansion [37,40] of round goby. However, there is an underrepresentation of studies that have systematically investigated mid- or long-term temporal changes of biological characteristics in *N*. *melanostomus* considering the different phases of an invasion (e.g. [17]). Better knowledge of round goby ecology over all phases of the invasion process is crucial since it can not only deliver estimates of associated ecosystem impacts [49] in this species, but also offers the chance to study general processes of invasion biology using this suitable model organism.

In the German section of the Danube River, which is one of the most important European long-distance dispersal routes for aquatic invasive species [50,51], *N*. *melanostomus* was first recorded in 2004 [52]. Six years later, round goby already comprised more than 50% of the total fish catch along the bank areas with densities of up to 20 individuals per square meter [19]. Brandner et al. [17] reported a female-biased invasion front with significantly larger body size and higher condition of round gobies at the invasion front compared to established, i.e. longer inhabited areas, postulating a ´bigger is better´ invasion strategy of this species. Those results were based on observing invasion performance along the fluvial gradient from total absence over first occurrence until establishment, but only included a time snapshot situation which questions the general validity of this pattern. Also in other studies, round goby populations at invasion fronts appear to be female-biased [17,22,53,54] with larger and faster-growing individuals [17,53], while established populations seem to be typically male-dominated (Trent River: [15,30]; Lake Ontario: [55]; Baltic Sea: [28]) with smaller individuals [53]. While downstream dispersal and range expansion in *N*. *melanostomus* are mainly governed by the drift of juveniles [56], upstream range expansion is seemingly not driven by out-migrating of weak or juvenile individuals that were forced to leave high density areas due to high intraspecific competition.

Instead, plasticity, e.g. of evolutionary and ecological determinants [13,17,38,57] may change life history strategies of invaders advancing from one phase to the next (e.g. [58]. Such factors need to be analyzed by systematically investigating population dynamics and effects from total absence until dominance of an IAS over longer time to understand long-term effects [59] and the importance of individual trait utilization [13]. The ongoing invasion of *N*. *melanostomus* in the upper Danube River–meanwhile decoupled from navigational vessel traffic—offered the opportunity to validate both the previously postulated ´bigger is better´ [17] and ‘individual trait utility’ [13] hypotheses by quantitatively studying early (introduction, establishment) and late (spread, impact) phases of a round goby invasion using both the spatial and the temporal fluvial gradient.

The objectives of this study were to (i) compare early and late phases of a round goby invasion at population- and specimen-level in a recently invaded lotic ecosystem using a fluvial gradient, (ii) validate phenotypic differences (length, weight and condition factor, hepato-somatic and gonado-somatic indices) between specimens representing those early and late population phases, (iii) analyze founder traits and demographic effects with respect to different points in time (before and after initial colonisation), considering abundance, sex ratio, parasitic load, feeding patterns, and to analyse (iv) local availability of benthic IAS as the most important food-resource. We hypothesized systematic differences in round goby between recently colonized and longer established areas concerning demography, morphology, feeding behavior, sex-ratio, parasitic load and thus test the validity of the ´bigger is better´ invasion strategy in the light of a possible competitive advantage of outlier specimens at the invasion front (i.e. validity of the ´individual trait utility´ hypothesis).

## Materials and methods

### Ethics statement

All investigations were carried out in accordance with the legal obligations of the Federal Republic of Germany and the local fishery law of Bavaria (´Bayerisches Fischereigesetz´ and ´Ausführungsverordnung Bayerisches Fischereigesetz´). Fish were caught under the permission of the local fisheries administration (´Fischereifachberatung Oberpfalz´, ´Fischereifachberatung Niederbayern´, ´Landratsämter´). Electrofishing was conducted under license number 31-7563/2 to the Aquatic Systems Biology Unit of Technische Universität München (TUM). In addition, all owners of the local fishing rights gave permission and supported sampling of *N*. *melanostomus* specimens. All required qualifications of the involved people (fishing licenses, electrofishing certificates, animal welfare training) were valid and formally approved. Based on the fisheries legislation (´AV BayFig´), all non-native fish had to be removed. Therefore all gobiids were euthanized (using an overdose of the anaesthetic MS-222 and immediately frozen to avoid degradation of gut contents) according to the German Animal Protection Law and the ordinance of slaughter and killing of animals (´Tierschlachtverordnung´). Live native fish captured by electrofishing were carefully returned to the river immediately after sampling. Since no experiments using living specimens were conducted, approval of the present study by a review board institution or ethics committee was not mandatory, yet the study concept was discussed and refined following advice from the TUM animal welfare and ethics committee.

### Field sampling

During each sampling campaign, i.e. in 2010, 2011 and 2015, the most recently invaded upstream border to which round gobies had reached in the upper Danube River was localized to determine the most actual “invasion front”. Hereby, round gobies were considered absent at a site where no individuals were caught at a minimum of 20 electroshocking minutes comprising multiple sampling points, analogously to the criteria defined by Brandner et al. [19]. The uppermost site where single individuals of *N*. *melanostomus* had been recorded (August 8^th^, 2015) was river km 2,443.8 (48°77'66.15"N; 11°60'16.54"E), just below the hydroelectric dam near the city of Vohburg, Germany. Here, single round gobies had been detected for the first time in 2014; thus this sampling site was named “invasion front 2014 (IF2014)”.

**Fig 1:**
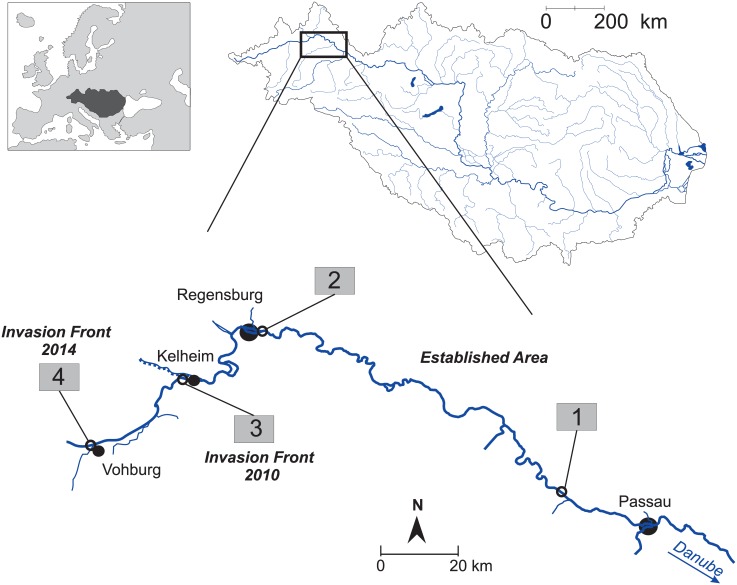
Study area at the upper Danube River between Austria and Germany. Study area with four representatively chosen rip-rap sampling stretches covering a recent round goby invasion along the headwater reaches of the upper Danube River in Bavaria, southern Germany. The consecutive numbers in grey rectangles denote two pioneering and two longer established (sub-)populations: a newly colonizing (sub-)population at a recent “invasion front 2014” (sampling stretch #4, first record: August 2014), a former “invasion front 2010” (sampling stretch #3, first record: September 2010) and two established sub-populations from an “established area” (sampling stretches #1 and #2, first record before 2007). The Danube basin and the location of the study area within the drainage area are highlighted. Filled black circles denote important cities along the Danube River.

The sampling design comprised a total of 420 point abundance sampling (PAS) points from fourteen samples of four representative river stretches (Fig 1, Table 1) with two longer established (sub-)populations from an “established area” where round goby had been recorded for the first time before 2007, and two pioneering populations from a former invasion front 2010 (“IF2010”), where a round goby invasion was observed in 2010, as well as the recent invasion front 2014 (“IF2014”).

**Table 1:** Sampling design and location of river stretches.

sampling design					first record	lower boundary		upper boundary	
#	stretch	river side	n	PAS		rkm	GPS	rkm	GPS
4a	Vohburg	left	1	30	2014^[1]^	2443.2	E 11°36'25.00" N 48°46'35.00''	2443.6	E 11°36'06.00" N 48°46'36.00''
4b	Vohburg	right	1	30	2014^[1]^	2443.4	E 11°36'16.00" N 48°46'35.00''	2443.8	E 11°36'12.00" N 48°46'40.00''
3a	Kelheim	left	3	90	2011^[1]^	2415.0	E 11°51'43.37" N 48°54'59.69''	2415.3	E 11°51'23.28" N 48°54'57.54"
3b	Kelheim	right	3	90	2010^[1]^	2412.7	E 11°53'28.79" N 48°54'26.74"	2413.0	E 11°53'14.52" N 48°54'28.36"
2b	Regensburg	right	3	90	before 2007^[u]^	2375.0	E 12°09'22.73" N 49°01'01.27"	2375.3	E 12°09'10.06" N 49°01'07.08"
1b	Vilshofen	right	3	90	2004^[2]^	2250.7	E 13°10'15.36" N 48°38'42.53"	2250.9	E 13°10'4.78" N 48°38'51.09"

Consecutive stretch number, stretch name, river side, total number of rip-rap samplings from 2010 to 2015 (n), total number of point abundance samples (PAS), first record of *N*. *melanostomus*, river kilometres (rkm) and GPS-coordinates of upper and lower boundaries (sorted in upstream to downstream order) of four representatively distributed rip-rap river stretches from both river shorelines along the upper Danube River.

^[1]^ own observations, ^[2]^ Paintner & Seifert [52], ^[u]^ = exact time point uncertain, but first recording clearly before 2007.

To exclude the effects of different mesohabitat structures on sex, size or other performance indicators, sampling exclusively focused on rip-rap structures (technolithal) which were previously found to be the preferred habitat of invasive round goby in the Danube River [19,60], representing about ⅔ of the available bank habitat in the study area. Since the dispersal of invasive gobies appears to be highly anisotropic following rip-rap banks along the river [40], samples from the different river sides were treated as independent samples. Thus, two spatially distant, representative rip-rap areas were chosen randomly to mirror the comparably “old” round goby population in the “established area” (stretches #1 and #2) where sampling was conducted at the right shoreline. Both shorelines were sampled at the comparably “young” populations IF2010 (stretch #3) and IF2014 (stretch #4) to increase potentially low catch numbers.

In addition to the screenings for localizing the invasion front in each of the sampling years, three main sampling campaigns were performed in 2010, 2011 and 2015 from August 30^th^ until September 30^th^, covering the late annual growth period of *N*. *melanostomus*. This narrow time window was chosen to allow comparability with previous studies (e.g. [17,40]) and to minimize any bias related to consideration of different lengths of growth periods. Fishes were collected during daylight from shorelines (in ~60 cm water depth) by PAS of electrofishing (ELT62-IID; Grassl GmbH, Berchtesgaden, Germany) with a duration of 10 s and a distance of 10 m between individual points, following [19]. Every shoreline sample comprised 30 PAS-points where all fishes were determined to species level, counted, measured (total length [*L*_T_] to nearest mm) and weighted (total mass [*M*_T_] to nearest 0.2 g). In general, *L*_T_ and *M*_T_ are important size- and growth-related performance indicators that determine fitness, food resource use and the likelihood of becoming prey. Sex of *N*. *melanostomus* was determined by an examination of the morphology of the urogenital papilla [29]. Since sex determination is unreliable for round gobies with *L*_T_ < 5 cm, this size class was classified as juveniles and excluded from sex-specific analyses. All fish species were inspected for infection rates with ectoparasitic plathyhelminths of the genus *Rossicotrema spp*. (black spot disease) and each specimen was assigned into four categories (0 = no black spots; 1 = few, i.e. < 5; 2 = medium, i.e. 5–100; 3 = many, i.e. > 100).

In addition to the demographic sampling for characterizations on the population level, individual traits of 150 round goby specimens were analysed on both river sides. This sample subset comprised a defined size-class (8–12 cm), to account for the fact that many morphometric indices assume isometry of body proportions in fish which can vary with size (e.g. [61]. The mean total length (*L*_T_) of all chosen specimens was 9.82 cm (SD = 1.15 cm).

The wet weights of liver, gut contents, ovaries in females, testes and seminal vesicles in males were recorded to the nearest 0.001 g. As round goby is known to serve as a paratenic host for acanthocephalans (e.g. [46], subadult acanthocephalans attached to inner organs were counted using a stereo-microscope. In order to test the “enemy release-hypothesis”, suggesting that invasive species carry less parasites in pioneering than in longer established or native populations (e.g. [62]), ecological indicators of parasite infection were applied according to Ondracková et al. [63], using mean abundance (i.e. mean number of parasites per host) and mean density (i.e. abundance per fish total mass).

To analyse spatial and temporal changes in water temperature regime, mean water temperature values of each day were used to calculate a mean water temperature for the sampling periods 2010, 2011 and 2014 at the measuring stations Neustadt (corresponding to sampling stretch #4) and Vilshofen (corresponding to sampling stretch #1). The raw-data, based on continuous measuring at both stations, are available from the Bavarian Environmental Agency (www.gkd-bayern.de). Mean water temperatures in the 4-week sampling periods of 2010, 2011 and 2015 at the two measuring stations strongly differed (2010: 14.4/14.5, 18.1/18.5, 2015: 16.1/16.9), but differences between sampling stretches #4 and #1 only ranged between 0.1°C (water gauge station Neustadt) and 0.8°C (water gauge Vilshofen) in each year, mirroring the slight warming-up in the river continuum. All of the sampling stretches were otherwise highly similar in terms of mesohabitat characteristics such as substrate (all rip-rap comprising the exact same bolder material and size), water depths (always 60 cm at all PAS points), flow velocity (<0.05 m/s in the preferred goby habitat above ground).

### Fish gut analyses

Digestive tract dissection, processing and fish gut analyses were conducted following Brandner et al. [19] with the anterior digestive tract being weighted to the nearest 0.001 g before and after emptying to obtain the wet weight of gut contents. All food items from the digestive tract samples were fixed in ethanol, identified to the lowest possible taxon considering manageable taxonomic levels, counted and visually estimated to the nearest % proportion of volume, using a stereo microscope.

### Benthic invertebrates

Quantitative samples of benthic invertebrates were collected using a suction sampler (as described in [19]) from the same sites where gobies were sampled (~60 cm water depth, duration = 120 s, three replicates). Altogether 60 samples of benthic invertebrates were preserved in 70% ethanol immediately after capture. A total of about 8,000 benthic invertebrates were identified to the lowest possible taxon considering manageable taxonomical levels. Organisms belonging to the same taxon or cumulative category were counted and expressed as catch per unit effort (CPUE [min^-1^]) following Brandner et al. [19].

### Indexing and statistical analyses

The somatic mass (*M*_S_) was calculated as *M*_S_ = *M*_T_−(*M*_indexed organ_ + *M*_g_) with *M*_g_ = gut content mass to compute the following indices: to test for differences in important body mass indices between specimens of a population, the hepato-somatic index (HSI = 100 *M*_liver_ *M*_S_^-1^) and the gonado-somatic index (GSI = 100 *M*_gonads_ *M*_S_^-1^) as a proxy of energetic investment into reproduction were calculated for both sexes [64]. Fulton’s condition factor *K* was calculated as *K* = 100 (*M*_T_—*M*_g_) *L*_T_^-3^ to assess length-weight relationships between populations and specimens [61]. To assess food uptake and to test for potential food limitation effects on feeding behaviour, the index of stomach fullness (*I*_SF_) was calculated following Hyslop [65] as *I*_SF_ = 100 *M*_g_ *M*_T_^-1^.

Analogously to Brandner et al. (2013a) the relative importance of a food item i among all items j for a population was calculated as the “index of food importance” (*I*_FI_):

$$I_{FI}(i)=100\;O(i)V(i){\left(\sum_{n=1}^{j}O(i)V(i)\right)}^{-1}$$

with O = % occurance of prey i and V = % volume of prey i

*I*_FI_ varies from 0 to 100, with higher values corresponding to a larger contribution of one food item as compared to total gut content. Since benthic invertebrate samples were treated like gut content samples, importance of naturally available prey was also calculated following the above mentioned formula as “index of environmental importance” (*I*_EI_) for each food item i.

Dissimilarity-distances (squared Euclidian distance) between the 14 samplings from the four river stretches were calculated using *L*_T_, *M*_T_ and *K* of females, males and juveniles, the proportions of females (as a relative sex ratio) and catch data (mean CPUE and frequency of occurrence (*f*_O_) of *N*. *melanostomus*, the most abundant autochthonous fish species barbel *Barbus barbus* (L., 1758) and chub *Squalius cephalus* (L., 1758) pooled as an indicator for abundant potential prey, and other fish species) from the corresponding rip-rap sampling sites as variables. The results were plotted as a two-dimensional non-metric multi-dimensional scaling (NMDS). In order to assess the importance of catch data, *L*_T_ and *M*_T_ as well as sex-ratio, additional NMDS analyses considering these factors separately were carried out.

As *L*_T_, *M*_T_, *K*, *I*_FI_, *I*_EI_, *I*_SF_, were not normally distributed (Shapiro-Wilk-test), multiple comparisons between populations and specimens were computed using non-parametric Kruskal-Wallis-tests followed by (post hoc) Bonferroni corrected Mann-Whitney-U pairwise tests. Differences from an expected equilibrium in the distribution of males and females as well as potential differences in the distribution of males and females (sex ratio) between the sampling areas were tested using the chi-square test. Significance was accepted at p≤0.05 for all statistical tests. Statistical analyses and plots were computed using PAST 3.06 [66].

Variation of round goby *L*_T_ and *K* was graphically displayed using boxplots. In concordance with Cerwenka et al. [13] goby specimens that deviated more than 1.5 times from the interquartile range were identified as outliers using PAST 3.06. To validate the outlying number of bigger and higher conditioned specimens 1,000 hypothetical allocations were estimated, following [67] using the extreme values as natural cutoffs. Subsequently, the mean number of outliers of the real-world and the estimated dataset were compared using a parametric t-test.

## Results

**Fig 2:**
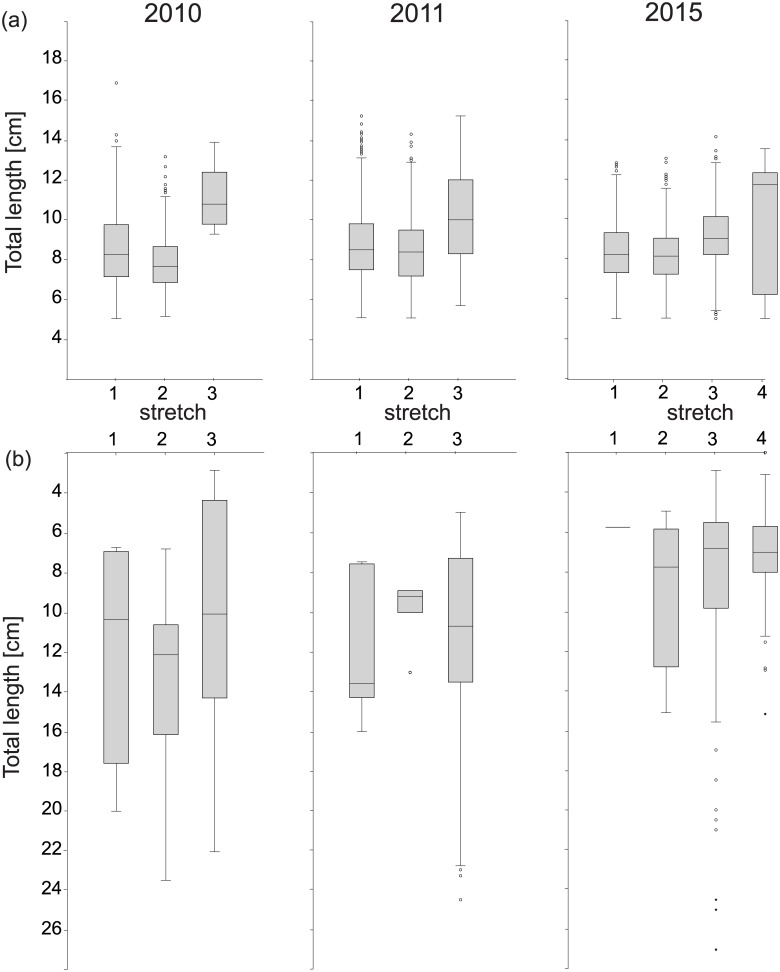
Total length of adult round gobies and the most frequent native fish species in 2010, 2011 and 2015 at four sampling stretches along the upper Danube River. Total length (*L*_T_ in mm) of a) adult invasive alien round gobies (*L*_T_ > 5 cm) and b) native barbel and chub at four sub-populations from the established (first record before 2007; stretches #1 and #2) and pioneering area (invasion front 2010 (stretch #3) and the invasion front 2014 (stretch #4)) in the upper Danube River, Bavaria southern Germany.

Throughout the different sampling years, comparatively low CPUE and low frequencies of occurrence of *N*. *melanostomus* were detected at the respective invasion fronts (Table 2), with by a factor of up to 30 greater densities at areas that were colonized since more than five years. Only in those areas with low densities of invasive gobies, native species such as barbel and chub were detected in frequencies of occurrence in a range of 50–80%, whereas values were only 2–25% in areas where frequency of occurrence of round goby exceeded 98%. In line with the “bigger is better” hypothesis, *N*. *melanostomus* specimens at the invasion front were significantly bigger in terms of total lengths and body masses compared to their conspecifics at longer inhabited areas (Fig 2 and Table 3, except for males at IF2014). More pronounced resource allocation into somatic growth is also reflected in greater condition factors at the invasion front, especially in females (Table 3). This is also supported by results on the level of specimens which had highest hepatosomatic and gonadosomatic indices at the invasion front, decreasing with increasing time since invasion (Table 4).

**Table 2:** Population dynamics in *N*. *melanostomus* and bycatch at three areas (stages) of the invasion.

first record	sampling area	year	PAS [n]	round goby		barbel & chub		other fish species	
				CPUE [PAS^-1^]	*f*_O_ [%]	CPUE [PAS^-1^]	*f*_O_ [%]	CPUE [PAS^-1^]	*f*_O_ [%]
August 2014	stretch #4 (IF2014)	2010	no data						
		2011	no data						
		2015	60	0.52	45.0	1.07	50.0	0.92	56.7
September 2010	stretch #3 (IF2010)	2010	60	0.08	8.3	1.28	68.3	0.90	53.3
		2011	60	1.63	63.3	2.05	78.3	2.00	63.3
		2015	60	8.42	100	2.23	60.0	1.33	68.3
before 2007	stretches #1& #2 (established area)	2010	60	5.82	100	0.02	1.7	0.18	13.3
		2011	60	7.15	100	0.00	0.0	0.25	10.0
		2015	60	8.57	98.3	0.02	1.7	0.78	25.0

The sampled rip-rap river stretches (upper Danube River, autumn 2009 to autumn 2015) were assigned to the three sampling areas “IF2014” (stretch #4), “IF2010” (stretch #3) and “established area” (stretches #1 and #2) using the time since invasion (year of first record), with the number of point abundance samples (PAS) and catch data of invasive *N*. *melanostomus*, *Barbus barbus* & *Squalius cephalus* (pooled) as most abundant autochthonous fish species and all other fish species. The catch (using electrofishing with continuous DC, duration 10s per PAS) is explained as the mean catch per unit effort (CPUE) [PAS^-1^] and the mean frequency of occurrence (*f*_O_) [%]. The abbreviation “nd” denotes “not detectable”. Data from the first time of occurrence are shown in bold.

**Table 3:** Comparison of performance indicators of *N*. *melanostomus* at population level (sampling 2015).

population-level	p	IF2014			IF2010			established area		
performance indicators		n	mean	SD	n	mean	SD	n	mean	SD
*L*_T females_ [cm]	***	10	**10.8** ^a^	2.8	238	**8.9** ^a^	1.3	228	8.1 ^b^	1.2
*L*_T males_ [cm]	***	12	**7.2** ^a^	2.6	137	**9.6** ^b^	2.0	200	8.5 ^c^	2.4
*L*_T juveniles_ [cm]	*	9	4.1 ^a^	0.5	130	3.7 ^b^	0.5	86	3.7 ^b^	0.6
*M*_T females_ [g]	***	10	**22.6** ^a^	14.3	238	**11.0** ^b^	5.0	228	7.9 ^b^	3.6
*M*_T males_ [g]	***	12	**8.8** ^a^	12.7	137	**14.8** ^b^	8.6	200	10.0 ^a^	7.3
*M*_T juveniles_ [g]	*	9	0.8 ^a^	0.3	130	0.6 ^b^	0.3	86	0.6 ^c^	0.3
*K*_females_	***	10	1.41 ^a^	0.34	238	1.46 ^b^	0.20	228	1.39 ^c^	0.13
*K*_males_	***	12	1.38 ^a^	0.15	137	1.43 ^b^	0.19	200	1.37 ^c^	0.16
*K*_juveniles_	ns	9	1.09 ^a^	0.31	130	1.19 ^a^	0.42	86	1.22 ^a^	0.59
females [%]		10	45.5		238	**63.5**		228	56.6	
males [%]		12	54.5		137	**36.5**		200	43.4	
overall sex ratio (f: m)	***	22	*1*: *1*.*20*		375	*1*: *0*.*56*		428	*1*: *0*.*88*	

Four sub-populations from the upper Danube River (sampling autumn 2015) were assigned to the categories “IF2014” (site #4), “IF2010” (site #3) and “established area” (2 sub-populations pooled: sites #1 and #2) using time since invasion (see Table 2). Numbers of fish analyzed, means and corresponding standard deviations (SD) of total length (*L*_T_), weight (*M*_T_) and Fulton´s condition factor (*K*) are displayed for both sexes and for juveniles (*L*_T_ < 5 cm). Percent females and males, as well as the overall sex-ratio were calculated from the total catch (excluding juveniles) of the sub-populations, respectively. Superscript letters denote significant differences (Kruskal-Wallis test) with p-values encoded by asterisks (*denotes p≤0.05; *** denotes p<0.001). Values highlighted in bold denote significant (Mann-Whitney U-test) differences between sexes. Values in italics denote significant (χ^2^ test) differences in the contribution of sexes between sampling areas.

**Table 4:** Comparison of performance indicators of *N*. *melanostomus* at specimen level.

specimen-level	p	invasion front 2014			invasion front 2010			established area		
performance indicators		n	mean	SD	n	mean	SD	n	mean	SD
**fecundity and condition**										
GSI _females_	ns	15	**0.81**	0.28	26	**0.67**	0.18	38	**0.72**	0.32
GSI _males_	ns	11	**0.35**	0.27	23	**0.23**	0.27	37	**0.26**	0.23
*K*_females_	***	15	1.54 ^**a**^	0.19	26	1.43 ^a^	0.16	38	1.34 ^b^	0.08
*K*_males_	***	11	1.51 ^**a**^	0.19	23	1.43 ^a^	0.20	37	1.34 ^b^	0.07
HSI _females_	***	15	**7.03** ^a^	0.68	26	**5.80** ^a^	1.74	30	**3.33** ^b^	1.02
HSI _males_	***	11	**4.79** ^a^	0.92	23	**4.28** ^a^	1.36	29	**2.87** ^b^	0.96
**feeding and prey-specific indices**										
*I*_SF_	ns	26	2.2	1.0	49	2.3	1.0	75	2.2	1.0
*I*_FI (EPT)_	**	26	0.87 ^**a**^	3.08	46	6.41 ^b^	20.2	72	0.09 ^a^	0.43
CPUE _(EPT)_ [min^-1^]	**	6	0.25 ^a^	0.27	18	1.67 ^b^	3.00	36	0.28 ^a^	0.47
*I*_EI (EPT)_	*	6	0.08 ^a^	0.12	18	1.58 ^b^	3.08	36	0.05 ^a^	0.09
**endoparasites (Acanthocephala)**										
abundance [n] females	***	15	84 ^a^	42	26	**142** ^b^	81	38	43^c^	48
abundance [n] males	***	11	82 ^a^	31	23	**92** ^b^	63	37	50 ^a^	36
density [n/g] females	**	15	2.8 ^a^	1.3	26	**10.6**^b^	6.7	38	3.6 ^b^	3.9
density [n/g] males	**	11	2.5 ^a^	0.5	23	**6.9** ^b^	4.7	37	2.7 ^c^	3.5
**ectoparasites (*Rossicotrema* spp.)**										
abundance [0–3] females	ns	15	0.0	0.0	26	0.04	0.20	38	0.0	0.0
abundance [0–3] males	ns	11	0.0	0.0	23	0.09	0.23	37	0.0	0.0

150 *N*. *melanostomus* specimens (mean *L*_T_ = 9.8 cm; SD = 1.2 cm) originating from the investigated sub-populations “IF2014”, “IF2010” and “established area” along the upper Danube River were sampled in autumn 2010, 2011 and 2015 for specimen specific analyses. 6 specimens had empty guts and were excluded from feeding and prey-specific analyses. Numbers of fish dissected, means and corresponding standard deviations (SD) of fecundity and condition indices (gonado-somatic index GSI, Fulton´s Condition Factor *K*, hepato-somatic index HSI), feeding indices (index of stomach fullness *I*_SF_; index of food importance of ephemeroptera, trichoptera and plecoptera *I*_FI (EPT)_) and prey-specific indices (catch per unit effort CPUE _(EPT)_ and index of environmental importance of ephemeroptera, trichoptera and plecoptera *I*_EI (EPT)_) and parasite infection indices were calculated for females and males. Values highlighted in bold denote significant differences (Mann-Whitney U-test) between sexes. Superscript letters denote significant differences (Kruskal-Wallis test) between populations with p-values encoded by asterisks (*denotes p≤0.05; ** denotes p<0.01; *** denotes p<0.001).

Specimens from the established area (n = 75) had a mean *L*_T_ of 9.82 cm (SD = 1.29 cm) with a slope of the length-weight-regression of b = 4.30 (R^2^ = 0.96; p<0.001). Specimens of the IF2010 (n = 49) had a mean *L*_T_ of 14.72 cm (SD = 5.42 cm) with a slope of the length-weight-regression of b = 4.61 (R^2^ = 0.87; p<0.001). Specimens of the IF2014 (n = 26) had a mean *L*_T_ of 12.40 cm (SD = 1.37cm) with a slope of the length-weight-regression of b = 8.73 (R^2^ = 0.84; p<0.001). ANCOVA comparisons of the slopes indicated no significant differences between these three groups (all p>0.05). However, in the “younger” populations, especially at the recent invasion front, an over-proportional number of large-growing individuals (i.e. outliers) led to an increase in the slope of the length-weight-regression up to nearly b = 9, thus strongly deviating from 3, and thus indicating non-isometric growth.

### Fish community

During the five-year sampling period, a total of 25 fish species (n = 4,398 individuals) were recorded in the upper Danube River, comprising 21 native and four non-native species. *Neogobius melanostomus* was by far the most abundant species, nearly contributing three quarters to the total catch (n = 3,224) and about 40% to the total biomass. In line with our hypothesis, the overall round goby sex-ratio significantly deviated from the expected equilibrium (χ^2^, p>0.001), with a greater than expected number of females (females:males = 1.31).

In the upper Danube River, non-native round goby was first detected in 2004 close to the city of Vilshofen (stretch #1) [52] and then successively invaded the upper reaches of this freshwater system: stretch #2 presumably in 2006, stretch #3 in autumn 2010 [17] (upstream dispersal ca. 35 river km / 4 years) and stretch #4 in 2014 (upstream dispersal ca. 30 river km within 4 years) (Table 1). In addition to round goby, also invasive Ponto-Caspian bighead and tubenose goby (0.6. % to the total biomass; 96 specimens) were found continuously, but at much lower and more varying abundances in the upper Danube River. Mean CPUE of bighead goby was 0.06 PAS^-1^ in 2010, 0.13 PAS^-1^ in 2011 and in the year 2015 it was 0.02 PAS^-1^. Tubenose goby was rarely found in 2010 (CPUE = 0.02 PAS^-1^) and 2011 (CPUE = 0.03 PAS^-1^), but its mean CPUE increased to 0.29 PAS^-1^ in the most recent sampling period. Racer goby, which was first detected in 2011 in the upper Danube River [68] was not caught during this study. Among autochthonous fishes (26.4% to to the total biomass; 870 specimens), the most abundant species were barbel (*B*. *barbus*) and chub (*S*. *cephalus*), together comprising about 12% to the total biomass (500 specimens). They showed highest abundance in river sections most recently invaded by round goby, i.e. stretch #3 (333 specimens) and stretch #4 (111 specimens).

Non-gobiid fish ectoparasite estimates varied between 0 (none) and 3 (many) and were highest at longer established sites, i.e. stretch #2 in 2010 (mean = 0.79) and stretch #1 in 2011 (mean = 0.67). In 2015, ectoparasite abundance (mean at stretch #1 = 0, #2 = 0.03, #3 = 0.08, #4 = 0) was lower than in 2010 and 2011. All differences were not significant (Bonferroni corrected Man-Whitney U, all: p>0.05).

### Round goby population data

Round goby was the most abundant fish species at the upper Danube River (stretch #1: n = 1,546; stretch #2: n = 1,037; stretch #3: n = 608; stretch #4: n = 33). Its mean abundance was positively and linearly related to time since invasion in all sampling years (2010: y = 1.63x, R^2^ = 0.53; 2011: y = 1.68x, R^2^ = 0.47; 2015: y = x, R^2^ = 0.34, all: p<0.001), yet at a rather high level of individual variability. Mean CPUE was highest at longer established sites and was significantly lower at the most recent pioneering population IF2014, in 2015 (Table 2). In females, *L*_*T*_ varied from 20 to 147mm, maximum *M*_T_ was 48.8g and maximum *K* was 2.89g*cm^-3^. In males, *L*_*T*_ varied from 20 to 169mm, maximum *M*_T_ was 64.8g and maximum *K* 1.97g*cm^-3^.

Population characteristics varied considerably with time since invasion. Relative round goby abundance increased by time since invasion: At the longer established (sub-)populations the mean CPUE was up to 8.5 PAS^-1^ (stretch #1) whereas it was much lower at the pioneering populations (Tab 2). Peak abundance of round goby at both, longer established populations and the IF2010, was 23 PAS^-1^. It was significantly (Bonferroni corrected Man-Whitney U, p<0.001) lower at IF2014 (3 PAS^-1^). The proportion of PAS points including invasive round goby (ʄ_Ο_) was significantly (Bonferroni corrected Man-Whitney U, p<0.001) different between stretches: ʄ_Ο_ was highest at pioneering (sub-)populations from the recently invaded areas and lowest at longer established ones (Tab 2). Sex-ratio (females:males) was female-dominated at longer established populations (stretch #1: 1.06, stretch #2: 1.65, stretch #3: 1.62) and differed significantly (χ^2^, p>0.05) from a theoretical equilibrium whilst it was balanced at the recent invasion front (stretch #4: 1.0). The proportion of juvenile round gobies decreased with time since invasion: juveniles were infrequently detected at longer established (sub-)populations (stretch #1: 13% and stretch #2: 20%), whereas medium abundance was found at the IF2010 (stretch #3: 25%) and a high frequency of occurrence at the IF2014 (stretch #4: 50%). Considering adults and juveniles, round gobies were significantly (Bonferroni corrected Mann-Whitney U test) larger at the IF2010 (stretch #3: median = 87mm) than at the established area (stretch #1: median = 81mm, p<0.001; stretch #2: median = 77mm, p<0.05). Sex-specific analyses revealed female round gobies to be significantly bigger at the recent invasion front than at all other sites (Table 3). Also, male round gobies were larger at the recent invasion front IF2014 than at all other investigated stretches (Table 3), however differences were less pronounced than in females and not significant (Bonferroni corrected Mann-Whitney U test, all: p>0.05). *M*_T_ of round goby varied between 0.2 and 64.8g, i.e. 1.0 and 48.8g in females and 0.5 and 64.8g in males. The heaviest females were found at the established area (i.e. stretch #1), whereas the heaviest males were detected at IF2010 (stretch #3). However, female round gobies were significantly (Bonferroni corrected Mann-Whitney U test, all: p<0.05) heavier at the most recent pioneering population IF2014 while male *M*_T_ was not significantly (Bonferroni corrected Mann-Whitney U test, all: p>0.5) different (Table 3). Fulton’s condition factor was significantly (Bonferroni corrected Man-Whitney U, p<0.001) lower at longer established populations than at IF2010 and lower than at IF2014 (Table 3). However, no clear sex-specific trend could be detected: females tended to have higher condition than males at the invasion front, but this difference was not significant (Bonferroni corrected Mann-Whitney U test, p = 1). Females also had a significantly (Bonferroni corrected Mann-Whitney U test, p<0.05) lower condition at the established area and the IF2010. Highest values were recorded at IF2014 in female (2.89g*cm^-3^) and male (1.97g*cm^-3^) round goby, indicating that ´individual trait utility´ seems to be a more powerful explanation for invasion success than a ´bigger is (always) better´ strategy.

Over a 5-year time period, at the IF2010 (i) the number of round goby (2010: n = 5, 2011: n = 98, 2015: n = 505), (ii) the peak abundance (2010: 1 PAS^-1^, 2011: 8 PAS^-1^, 2015: 17 PAS^-1^) and (iii) the proportion of juveniles [%] (2010: 0, 2011: 2, 2015: 28) increased continuously. On the other hand, here the sex-ratio became less female-biased (2010: 4.0, 2011: 1.23, 2015: 1.73), the ʄ_Ο_ [%] (2010: 92, 2011: 37, 2015: 0), mean *L*_*T*_ [mm] (2010: 113, 2011: 101, 2015: 77; Fig 2) and the mean condition [54g*cm^-3^] (2010: 1.54, 2011: 1.53g, 2015: 1.38) decreased from the initial invasion in 2010 until 2015. Excluding juvenile round gobies, both female and male individuals were significantly (Bonferroni corrected Mann-Whitney U test, all: p<0.001) larger at IF2010 than in the established area five years after invasion (Tab 3).

**Fig 3:**
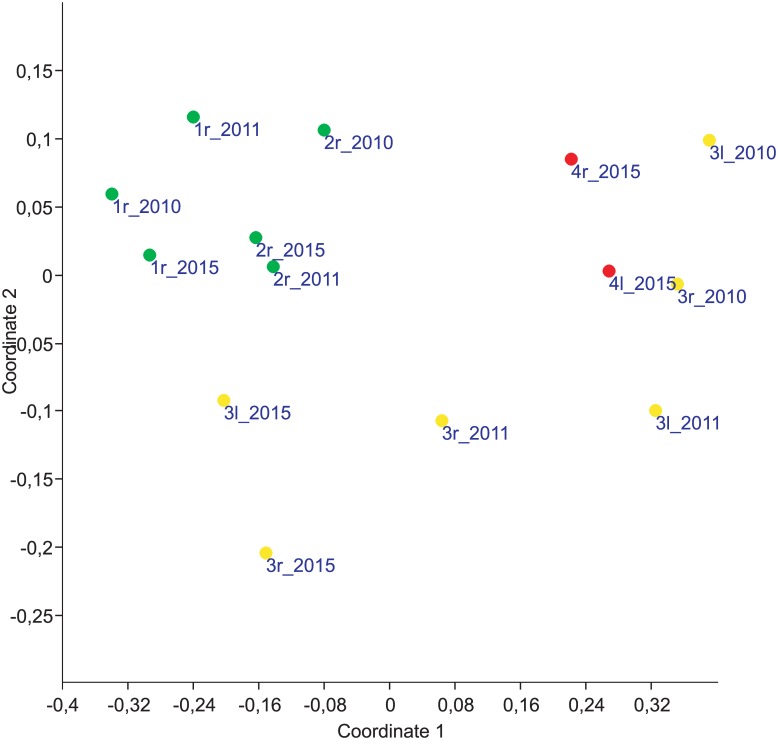
Nonmetric multidimensional scaling of *N*. *melanostomus* performance metrics. Nonmetric multidimensional scaling (NMDS) of *N*. *melanostomus* population-specific performance metrics calculated from point-abundance sampling data (autumn 2010, 2011 and 2015) in the upper Danube River, Bavaria, southern Germany. Dissimilarity-distances between 14 samples from four river stretches were calculated using the squared euclidian distance and displayed in green (“established area” with stretches #1 and #2), yellow (“invasion front 2010”, stretch #3) and red spots (“invasion front 2014”, stretch #4). The spot labels are encoded with sampling stretch number, river side (r = right, l = left) and year of sampling. *L*_T_(f), *L*_T_ (m), *L*_T_(j), *M*_T_(f), *M*_T_(m), *M*_T_(j), *K*(f), *K*(m), *K*(j), proportion of females and catch data (mean CPUE and frequency of occurrence of (i) *N*. *melanostomus*, (ii) *Barbus barbus* and *Squalius cephalus* (combined) and (iii) other fish species) from the corresponding sampling sites were used as variables (stress = 0.05).

An analysis of *N*. *melanostomus* population-specific performance metrics from PAS-data using NMDS resulted in structuring the 14 samples by time since invasion with a separation of invasion front samples from longer established ones (Fig 3): Obviously, the longer established (sub-)populations (stretches #1 and #2, green spots) were separated from both the most recent pioneering (sub-)populations from IF2014 (stretch #4, red spots) as well as from the early IF2010 samples.

Interestingly, the latest samples from IF2010 (stretch #3) were more similar to the samples from the longer established area than to those from the recent invasion front: together, all samples from IF2014 (red spots) and the early samples of IF2010 (yellow points with positive values at coordinate1) strongly differed from a distinct second cluster (hierarchical cluster-analysis using Ward´s method and squared Euclidian distance as similarity measure–not shown herein) consisting of all established area samples (green spots) plus the latest samples from IF2010 (yellow spots with negative values at coordinate 1). Since those latest samples from IF2010 were stronger associated with the samples from the established area, this pattern indicates a less important spatial influence vs. a pronounced temporal effect. Moreover, this pattern seemingly tends to disappear in a short time-span after initial colonisation. This may mirror whether a fading-out of the driving forces or the disappearance of outlier specimens by time and thus underlines the fast pace of underlying running processes (changes) in the early phases of a biological invasion. Analogously to Brandner et al.[17], the factors mainly accounting for the observed separation by time (since invasion) were catch data, *L*_T_ and *M*_T_, whereas the sex-ratio only played a minor role.

### Round goby specimen data

Female specimens had highest GSI at the IF2014 (median = 0.83) and relatively lower GSI at IF2010 (median = 0.63) and longer established populations (stretch #1: 0.59, stretch #2: 0.75). However, differences were not significant (Bonferroni corrected Mann-Whitney U test, all: p>0.05). In male round gobies no significant differences in GSI (Bonferroni corrected Mann-Whitney U test, all: p>0.05) were detected but GSI was highest at the IF2014 (0.23) and at established area stretch #1 (0.27). Also, it was about two times lower at IF2010 (0.12) and established area stretch #2 (0.14). HSI increased significantly (Bonferroni corrected Mann-Whitney U test, all: p<0.01) with time since invasion from stretch #1 (median = 2.46) to stretch #2 (median = 3.72) and stretch #3 (5.01). Highest HSI was observed at the IF2014 (stretch #4: median = 6.37) but differences were not significant (Bonferroni corrected Mann-Whitney U test, p>0.05). No significant differences were found for the ISF between stretches (Bonferroni corrected Mann-Whitney U test, all: p>0.05). ISF altered between 1.9 (stretch #4) and 2.4 (stretch #3) and was intermediate in the longer established populations (median stretch #1 and #2: 2.0). In general, females had both significantly higher GSI and HSI at all sampling stretches and a significantly higher endoparasite load at IF2010 compared with their male conspecifics (Table 4).

Analogously to Brandner et al. [17], abundance and density of acanthocephalan parasites also differed in round gobies along the upper Danube in 2015. Abundance was highest at the IF2010 (median = 99,) and at the IF2014 (median = 88) and was significantly (Bonferroni corrected Mann-Whitney U test, all: p<0.01) lower at longer established populations (median: stretch #1 = 53, stretch #2 = 9). Density of acanthocephalan parasites showed comparable results being highest at IF2010 (stretch #3) and lowest at longer established populations (median stretch #1 = 4.47, #2 = 0.66, #3 = 6.83, #4 = 2.68). All comparisons were significant (Bonferroni corrected Mann-Whitney U test, all: p<0.05). No sex-specific differences were found in acanthocephalan parasite abundance and density at any of the analyzed stretches (Bonferroni corrected Mann-Whitney U test, all: p>0.05).

Regarding the food consumption as an indicator for trait utility, round goby feed was more diversified at the pioneering (sub-)populations (i.e. IF2014) than at the established area in 2015: At IF2014, round goby consumed various invertebrate species to a relatively high extent (IFI [%]: *Chelicorophium* spp. = 24.1; Gastropoda = 22.4; *Dikerogammarus* spp. = 21.3; Amphipoda = 21.5), only Amphipoda (IFI [%]: stretch #1 = 35.3; stretch #2 = 17.4) and *Dikerogammarus* spp. (IFI [%]: stretch #1 = 9.0; stretch #2 = 35.3) had increased IFI-values at the established area. The IFI at IF2010 changed considerably over the sampling period: at the beginning of this study 2010 bivalves (34.6%), gammarids (*Dikerogammarus* spp.: 32.7%) and amphipods (*Chelicorophium* spp.: 27.0%) had highest IFI values, whereas in 2015 round goby mainly consumed *Chelicorophium* spp. (32.0%), supporting the influence of ´individual trait utility´.

### Round goby individual trait utility

At the population level, we found n = 100 (6.2%) round goby individuals distinctively deviating in *L*_T_ or *K*: an outlying large body-size was recorded in n = 40 (2.5%), an outlying *K* in n = 64 individuals (4.0%; high and low each n = 32, 2%). A sex-specific trend of invasive alien gobies at an invasion front was described for IF2010 [17], but was not apparent at the most recent pioneering (sub-)population (Bonferroni corrected Mann-Whitney U test, p = 1). However, females tended (Bonferroni corrected Mann-Whitney U test, p>0.05) to have a higher condition than males in pioneering (sub-)populations and vice versa at the established area (Bonferroni corrected Mann-Whitney U test, p<0.05).

On the specimen level, we found eight individuals (9.2%) carrying outlying traits, i.e. one individual (1.1%) with an outlying high number of acanthocephalan parasites, and one female (2.2%) and six male individuals (14.3%) with higher GSI. The number of these individuals having such traits was not significantly (t-test *p* > 0.05) different from the hypothetical mean of 1000 estimations for the GSI of male individuals, significantly lower (t-test *p* < 0.001) for acanthocephalan parasites, and could not be calculated for female GSI because of too little variance of estimated values. Occurrence of individuals with outlying traits was independent from the sampling area and thus from time since invasion (χ2-test, p = 1): only one specimen (a male round goby with an elevated GSI) originated from a recently pioneering (sub-)population.

### Food resources

Non-native amphipod species of Ponto-Caspian origin, i.e. *Dikerogammarus spp*., *Chelicorophium spp*., *Jaera spp*., were the most abundant invertebrate species in the upper Danube River. Their volumetric proportion to the total sample content (n = 60) varied between 0% and 80%, and per river stretch it varied between 12% (stretch #2) and 24% (stretch #4). Invertebrate IAS contributed more than 40% to total environmental samples (*I*_EI_ = 83%) whereas indigenous species only had a proportion of about 10% (*I*_EI_ = 2%). Alien and native organism distribution was not significantly different within the upper Danube River but at stretch #1 IAS significantly contributed strongly to *I*_EI_ (Bonferroni corrected Mann-Whitney U test, p<0.01) whereas stretch #2 was dominated by native organisms (Bonferroni corrected Mann-Whitney U test, p<0.05). Molluscs (i.e. *Dreissena spp*., *Corbicula spp*., *Potamopyrgus spp*.) were of minor importance in the entire investigated area, summing to 2% of total sample content. In contrast, all Ephemeroptera, Trichoptera and Plecoptera (EPT), native to the upper Danube River and typical for the autochthonous fauna, occurred at very low densities only and no significant (Bonferroni corrected Mann-Whitney U test, p<0.01) differences in species abundance was found between sampling stretches. However, EPT were positively selected by preferential consumption of invasive round gobies in the upper Danube River, except for stretch #1 were no EPT-Taxa were found in environmental samples.

## Discussion

In line with the initial hypotheses, proposed for the first time in Brandner et al. [17] and Cerwenka et al. [13], the results of this study generally confirm previous findings of both the ´bigger is better´ and the ´individual trait utility´ invasion patterns in round goby. After a further upstream movement of the invasion front of about 30 river km within four years, the finding that round goby pioneering populations significantly differ from those from longer established areas has been reproducibly confirmed. Specimens from recently colonized areas were on average bigger (larger and heavier) and had a higher body condition compared to their conspecifics from established areas, where intraspecific competition is stronger and food choice thus more limited. The over-proportional number of non-isometric large-growing individuals at recently founded (sub-)populations cannot be caused by size effects since the specimen-samples had been size-class-selected, underlining our ´individual trait utility´-hypothesis. In addition, single individuals are characterized by outliers in selected traits (body size and condition) and might deliver over positional support to this species invasion success: In concordance with the trait utility hypothesis of Cerwenka et al. [13], distinctively deviating round goby individuals (n = 100, 6.2% of all individuals) having an outlying large body size (n = 40, 2.5% of all individuals) or an outlying condition (n = 64, 4.0% of all individuals) were predominantly recorded at pioneering (sub-)populations of both years. 20% (IF2010) and 27% (IF2014) of all individuals at the pioneering (sub-) populations had an outlying high *L*_T_ or *K* compared to their conspecifics from the established area. Individuals with highest *K* values were recorded at IF2014 in both sexes: females (2.89g/cm^3^) and males (1.97g/cm^3^).

Analogous to these findings from the upper Danube River, such ´bigger is better´- and therein ‘individual trait’-patterns have been reported from other newly invaded ecosystems worldwide (Table 5), suggesting that it can be considered a generally valid determinant of early-phase invasion patterns in invasive round goby (*Neogobius melanostomus*) and presumably in other invaders, too (e.g. [14,69]).

**Table 5:** Comparison of *N*. *melanostomus* first record data.

first record data	river, location	*L*_T_ [cm] (mean ± SD)	sex ratio (f:m)	n
Paintner & Seifert (2008)	Danube, Passau	11.0 ± 1.5	no data	43
Kalchhauser et al. (2011)	Rhine, Basel	9.2 ± 1.0	1:0.3	11
Hempel & Thiel (2013)	Elbe, Hamburg	18.9	0:1	1
Gutowski & Fox (2011)	Trent River, Ontario (Canada)	8.1 ± 0.2 (f) 9.1 ± 0.2 (m)	1: 2.2	172
Brandner et al. (2013)	Danube, Kelheim	10.4 ± 2.3 (f) 10.2 ± 2.6 (m)	1:0.77	106
Schomaker & Wolter (2014)	Oder, Friedrichsthal	10.0 ± 2.7	1:0.4	7
this study	Danube, Vohburg	7.8 ± 3.7	1:1	33

Six datasets of *N*. *melanostomus* first recordings containing ´bigger is better´-patterns from several fluvial ecosystems. Mean *L*_T_ and SD, sex ratio and number of recorded specimens were re-calculated and re-formatted in case of not being explicitly presented in these studies to obtain comparability of data. Although different sampling methods were applied therein, these studies commonly reported large sized individuals from recently colonized habitats (“invasion front”). Note that the fish sampling of our study took place one year after *N*. *melanostomus* first record.

As a result of potentially increasing intraspecific competition, longer established populations were clearly female-dominated (seemingly heading to a 1:2 proportion in m:f), comprised smaller sized, less heavier individuals with lowest condition and lowest hepato-somatic index. The proportion of juveniles seems to rise with time since invasion, being highest at longer established (sub-)populations. In addition to these demographic determinants, differences in morphology, feeding, behaviour and parasitic load on the individual level support the suggested great plasticity in this species [13,17,22,38]. However, an accumulation of individuals with particularly fitting traits at pioneering sites could only be verified partially: differences are supposed to be small at the level of single individuals and thus a high number of analyzed specimens would be needed to explain a linkage of invasion success and the ´individual trait utility`hypothesis.

In contrast to earlier findings by Brandner et al. [17], a much lower parasite load in the specimens from the most recent pioneering (sub-)population IF2014 compared to earlier years was evident in this study, questioning that the greater availability of parasite-infected intermediate hosts at lower goby densities would generally result in a violation of the ´enemy release hypothesis´ in this species. Instead, habitat-dependent local conditions affecting the number of infected intermediate hosts seem to play a more important role than previously expected.

**Fig 4:**
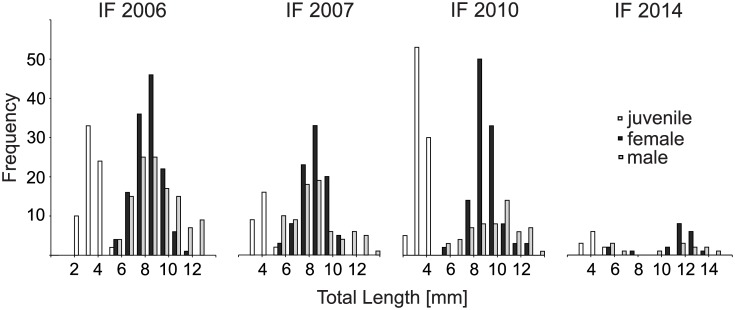
Spatio-temporal invasion performance: *Neogobius melanostomus* along the upper Danube River. Spatio-temporal performance of four *Neogobius melanostomus* (sub-)populations (stretches #1 to #4) in the upper Danube River displayed by length-frequency histograms (white bars = juveniles, grey bar = females, black bars = males). Fish data are based on point abundance sampling of electrofishing in autumn 2015 in the upper Danube River, Bavaria, southern Germany. The time since invasion in years is displayed within each panel in brackets and means the difference between sampling time and first record.

Another important difference between the results of this study and previous findings is that the sex ratio in gobies at the most recent pioneering (sub-)population IF2014 was more or less equilibrated, whereas a higher number of females was expected from the previous assessments. Most likely, this difference can be explained by the invasion front having reached a major hydroelectric dam in the Danube which is a major barrier for the further dispersal of fish, and thus slowing down the further upstream movement and resulting in an accumulation of specimens in this area. Second, the time of sampling may also play a role since the sampling at IF2014 was conducted one year after the first appearance of gobies in this area, diluting the invasion front effect at this site, since in the second year after first recording, reproduction has already been started (Fig 4). Consequently, the absence of the previously described sex bias at invasion fronts cannot be excluded based on our dataset. On the other hand, the migration barrier is likely to not stop but only delay the further upstream dispersal of gobies at this site since the upstream habitat quality is similar in terms of rip-rap bank habitat structures and temperature regimes, and since a fish bypass channel provides a selective opportunity for further upstream-directed migration.

### Assessing propagule

In recent years, several studies have underlined the key importance of propagule pressure in the invasion success of non-native species [12,70], whilst specific characteristics of propagule itself have hardly been examined.

According to our results, larger individuals with greater body condition and greater energy reserves are likely to be the specimens which act as “prime emperors” with the ability to pushing an invasion front forward. This seems reasonable, since *N*. *melanostomus* does not possess a great swimming ability, however manages to perform upstream directed dispersal rates of 7–10 river km per year in the upper Danube. This may mirror the natural dispersal rate without any transportation vectors being involved, since industrial ship traffic ends shortly below stretch #3 by entering the Rhine-Main-Danube-Canal. At the same time, the greater availability of food resources for individuals in recently colonized areas with lower intraspecific competition can also contribute to the same finding. Whilst it cannot be delineated from our data which of those two mechanisms is more important, the same pattern with similar effect sizes between recently colonized and established populations has been evident in the upper Danube river for several years (2009, 2010: Brandner et al. [17]; 2014: this study), supporting the theory that this pattern is stable over time and rather independent from habitat variability as well as anthropogenic migration vectors via industrial shipping which only affects the downstream areas.

**Fig 5:**
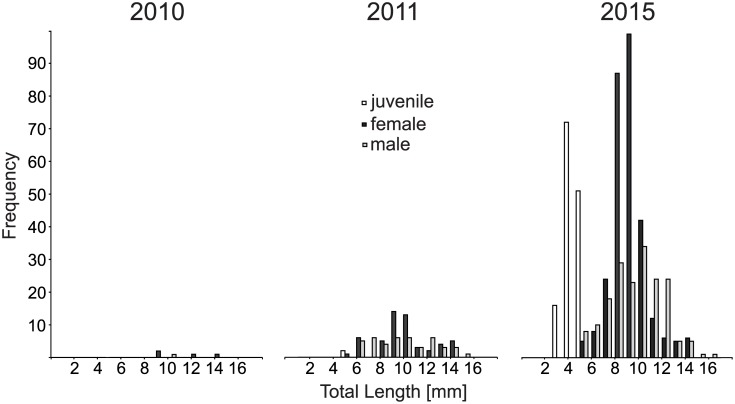
Five years of invasion–Temporal population dynamics of *Neogobius melanostomus* at the invasion front 2010. Five years of invasion at the invasion front 2010 (IF2010): Length-frequency-histograms displaying the temporal dynamics of the *N*. *melanostomus* population at stretch #3 from first record (introduction, 2010) to first appearance of juveniles, i.e. successful reproduction (establishment, 2011) until mass development (spread, 2015) with white bars = juveniles, grey bar = females, black bars = males. Fish data are based on point abundance sampling of electrofishing in autumn 2010, 2011 and 2015 in the upper Danube River, Bavaria, southern Germany.

Consequently, in *N*. *melanostomus*, the underlying ´*introduction effort*´ seems to consist of a relatively small inoculation size of some individuals with particular traits, potentially increasing the locally adaptive trait of a pioneering (sub)-population or instead, a relatively high propagule number of constantly migrating specimens (Figs 4 and 5).

### Stages of the invasion process and management implications

Long-term observations of the round goby invasion in the upper Danube River can be used to differentiate the stages of the invasion process based on the different sampling time points at fixed sampling locations (Fig 5). The sampling design used herein offered the chance of such an analysis. Since the ability to assess time since invasion (in other words: the age of an IAS population) could be crucial to predict potential success in IAS management, such analyses are important and urgently needed tools not only for scientists, but also for the practical management of an invasion itself. To date, very few systematic (long-term) sampling points exist in the Danube and in other river systems that would allow rigorous analyses of invasion processes based on consistent sampling strategies. The value of establishing more of such sampling sites is exemplarily illustrated by this study.

At the pioneering population IF2014, one year after first recording, the local *N*. *melanostomus* (sub-)population has managed to proceed from the initial “introduction” to the phase “establishment”. Here, the invasion process has been started by the introduction of few quite “aberrant”, i.e. large and best conditioned pioneering individuals (prime emperors), supporting the ´bigger is better´-strategy. Here also large gobies and two cohorts of juveniles can be observed (Fig 4), the missing medium size class (i.e. missing migrating size class) underlines the ´bigger is better´ range expansion hypothesis from our earlier study [17] and the importance of juveniles with the ability to massive downstream drift in neogobiid invasion processes [56]. Since successful reproduction has already begun here, potential eradication measures using egg-traps as suggested by Hirsch et al. [71] or reducing propagule by hook-and-line methods have become uncertain, closing the window for reasonable management measures in time.

At the pioneering population IF2010, five years after the first record of alien invasive round goby, massive reproduction has led to a significant increase in population density with a high proportion of juveniles (“boom”-scenario). Since no indicators of resource limitation can be observed (still comparably high HSI), this seemingly mirrors the “spread”-phase indicating that the “impact” phase has not been reached, yet. Here, *N*. *melanostomus* invasion is running at high performance, making potential reasonable management trials nearly impossible.

Seemingly, longer established populations from stretches #1 and #2 (established area) have already reached the “impact” stage, since important performance indicators (e.g. *L*_T_, *M*_T_, *K*; Table 3) and feeding and prey-specific indices (e.g. HSI; Table 4) mirror sub-optimum conditions for growth and a beginning limitation of environmental variables (e.g. *I*_EI (EPT)_). Although this sub-population has still increased over time (Table 2) and no significant differences in the *I*_SF_ among sub-populations could be observed, an ongoing onset of a food resources limitation could still be masked by the pronounced generalistic feeding abilities in this species [19]. Such an interspecific competition-related impact has also been reported from the Hudson River estuary, where the appearance of the invasive alien zebra mussel *Dreissena polymorpha* was followed by steep declines (65–100%) in population size of all species of native bivalves within 10 years [72]. Moreover, after initial declines, populations of native bivalves have meanwhile stabilized or even recovered due to increasing intraspecific competition among the IAS. Analogously, our study from the upper Danube River provides evidence for parallel processes between invasive *N*. *melanostomus* and indigenous fishes and benthic invertebrates: after initially strong individual and population growth, coupled with a decline in native species, a second step of increased intraspecific competition and predation leads to lower growth, in turn providing a survival chance for the native competitors. Especially the fact that *N*. *melanostomus* invasions appear to be very fast running processes, with minimum duration of only one year from first introduction until establishment, only prevention of introductions can be considered a promising and reasonable management approach.

### Genesis of a novel food web

Interactions between highly altered river systems and IAS often result in novel food webs and ecosystem structures [73,74], which comprise new combinations of species in habitats that are very different from those in the original habitats [75,76]. Without a doubt, every ecosystem was novel at one time, reflecting both the difficulty of precisely defining ecosystems and the fact that no place on earth is static, however what is different today, is the fast pace at which such complex change happens [77]. Such phase shifts are irreversible, driven by the creation of new assemblages with increasing numbers of interacting organisms, comprising previously isolated species from Asia, North-America, New-Zealand and the Ponto-Caspian region that have been introduced to the upper River Danube over the last two decades [19,26,78]. This phase shift is to date not reflected in the conservation management of this system. For instance, restoration targets for the fish fauna in the context of the European Water Framework Directive consider the historic reference state as a primary benchmark which is highly unrealistic to be achievable in light of novel biological community structures [79,80]. Looking closely to the edges of their distribution, ongoing upstream directed range expansions for several benthic aquatic IAS become obvious for the upper Danube River [81]. Among alien invasive amphipods and molluscs, the most important goby prey items (*Dikerogammarus villosus* Sowinsky; *Dreissena polymorpha* Pallas, *Corbicula fluminea* Müller, 1774) [19] have already colonized further upstream sections of the upper Danube River [81], therefore it just seems to be a question of time until also first individuals of *N*. *melanostomus* will further invade the upper Danube River.

Considering the recent finding, that synergistic impacts by invasive *D*. *villosus* and *N*. *melanostomus* can explain native gammarid extinction [82], significant impact on native gammarids must be expected after the arrival of *N*. *melanostomus*. Consequently, especially the success of Ponto-Caspian invaders apart from the navigational routes in upstream sections of the Danube River reflects fundamental ecological changes in the large European freshwater ecosystems [17], which make a return to original communities almost impossible [79]. In case of few pioneering individuals taking advantage from novelties due to alternative adaptation, outlier-specimens may possibly also drive -or even accelerate- the genesis of novelties themselves. Thus, an ´indiviudal trait utility´ could possibly play a yet underestimated part in complex environmental change such as the genesis of a novel food-web.

## Conclusion—Is bigger really better?

The recent upstream-directed colonization along the fluvial gradient of the upper Danube River with its distinct invasion front offered the unique possibility to validate our earlier findings and to further study determinants of biological invasions in one of the most successful aquatic invasive alien species worldwide, the round goby.

Non-random, specific differences between newly introduced and longer established populations in *N*. *melanostomus* point to generally valid changes by time during an invasion and in an IAS itself.

Primarily, large sized pioneering invaders with increased exploratory behavior [57], high phenotypic plasticity [38] and an increased competitive ability [17] seem to act as (prime) emperors of new habitats, hereby strongly following man-made river-bank structures [40]. Thus, bigger specimens seem to be a definite characteristic of a round goby invasion front. This finding confirms the bigger is better invasion strategy and underlines the importance of the ´individual trait utility´-hypothesis. However, Cerwenka, et al. [13] did not find strong trait distribution differences between such individuals, comparing recently invaded and longer established sites. This paradox may lie (i) in fast personality-dependent dispersal [83] since high migration rates may rapidly level out small scale trait distribution differences even across large distances [40,47] or (ii) in multiple inoculations, which may have the same leveling effect by increasing the likelihood of introducing locally fit individuals [13].

It thus remains open, whether already large-sized individuals act as prime emperors from downstream located areas (bigger is better) or if newly arriving specimens alternatively just benefit from low (intraspecific) competition, a lower predation risk and a less limited availability of food resources at the expanding edges of their population distribution. Consequently, in the light of the “individual trait utility hypothesis” [13], it does not seem unrealistic that “alternative performers” are the most likely candidates to arrive and benefit from ideal conditions for growth in newly invaded areas.

In conclusion however, both the herein validated ´bigger is better´ strategy [17] as well as the ´individual trait utility´ hypothesis [13] appear to be state-of-the-art explanations for round goby invasion success to date.
